# Oily wastewaters treatment using *Pseudomonas* sp. isolated from the compost fertilizer

**DOI:** 10.1186/2052-336X-12-77

**Published:** 2014-04-28

**Authors:** Abooalfazl Azhdarpoor, Bagher Mortazavi, Gholamreza Moussavi

**Affiliations:** 1 Department of Environment Health Engineering, Faculty of Health, Shiraz University of Medical Sciences, Shiraz, Iran; 2 Department of Environment Health Engineering, Faculty of Medical, Tarbiat Modares University, Tehran, Iran

**Keywords:** *Pseudomonas*, Oil, Wastewater, Lipase, Bacteria

## Abstract

**Background:**

Discharging the oily wastewater in the environment causes serious problems, because of the oil compounds and organic materials presence. Applying biological methods using the lipase enzyme producer microorganisms can be an appropriate choice for treatment of these wastewaters. The aim of this study is to treat those oil wastewaters having high concentration of oil by applying lipase enzyme producer bacteria.

**Materials and methods:**

Oil concentration measurement was conducted using the standard method of gravimetric and the wastewater under study was synthetically made and contained olive, canola and sunflower oil. The strain used in this study was *Pseudomonas* strain isolated from compost fertilizer. The oil under study had concentration of 1.5 to 22 g/l.

**Results:**

The oil removal amount in concentrations lower than 8.4 g/l was over 95 ± 1.5%. Increase of the oil's concentration to 22 g/l decreases the amount of removal in retention time of 44 hours to 85 ± 2.5%. The best yield of removing this strain in retention time of 44 hours and temperature of 30°C was achieved using Ammonium Nitrate as the nitrogen resource which yield was about 95 percent.

**Conclusion:**

The findings of the research showed that *Pseudomonas* bacteria isolated from the compost fertilizer can degrade high concentration oils.

## Background

Oily wastewaters produced in oil factories provide various environmental problems, because of having different pollutant compounds [1,2]. Reduction of the surface water's oxygen and its effect on the aquatic organism strains are among the problems [3]. Various physical, chemical and biological methods have been used to remove such compounds [4,5]. Cheryan and Rajagopalan show that the conventional treatments such as gravity separation, air flotation, coagulation are not efficient to solve this problem, especially when the oil droplets diameters are less than 20 μ. Fischer et al. worked on a combination of gravity separation and a downstream microfiltration. Sarakulski et al. proposed to treat oily wastewater by a combination of ultrafiltration and reverse osmosis processes. However, for real wastewaters is observed a membrane fouling or a high regeneration frequency [6].

In biological methods, the microorganisms with high enzyme activities can degrade oil. Lipase is an enzyme which can degrade fats into glyceride and fatty acids [7]. Many microorganisms including fungi, yeast and bacteria are able to produce lipase enzyme. They have been studied in many researches. *Penicillium*, *Yarrowia*, *Geotrichum*, *Bacillus, Acinetobacter* and *Serratia* are samples of these microorganisms, among which bacteria are more applied in oily wastewaters treatment [8-10]. Dongzhi *et al.,* worked on construction of a whole-cell catalyst displaying a fungal lipase for effective treatment of oily wastewaters. They declared that 96% of oil (5 mg/l oil) and 97% of COD were removed [11]. Lan et al. investigated biodegradation of oil wastewater by free and immobilized *Yarrowia lipolytica.* Their results showed that immobilized *Y. lipolytica* might be applicable to a wastewater treatment system for the removal of oil [12]. Bacteria which are able to produce lipase can be found in various places, including dairy industries and oil wastes, hot springs and soils contaminated with oils [13,14]. Hasanuzzaman et al. separated a novel, oil-degrading bacterium from a hot spring in Japan. The 16S rRNA gene sequence analysis revealed it as a new strain of *Pseudomonas*[15]*.* Selva et al., examined isolation of lipase-producing *Bacillus* strains from the soil sample of coconut oil industry. Results indicated that the lipase activity was maximum (2.2U/ml) for *Bacillus* sp. in 1.5% concentration [16]. Adding lipase-producer bacteria into the biological treatment units can increase the yield of oily wastewater treatment systems [17]. In fact, these bacteria speed up the treatment process through fat degradation. In this study, strains were isolated from compost fertilizer obtained from a solid waste disposal plant. Various researches have shown that compost fertilizer is suitable for isolating the resistant bacteria with a high ability to decompose organic compounds. Anna et al. (1998) showed that methanotrophic bacteria isolated from compost successfully converts CFCs into simpler products [18]. In other experiments, Ghazifard et al. (2001) reported the isolation of heat resistant microorganisms from a composting mass [19]. Since the compost fertilizer is suitable for the growth of various microorganisms which are able to degrade resistant compositions and since the related bacteria have been in contact with various oil combinations (edible and industrial) and on the other hand, there has not been any similar research on degrading the oil combinations by the lipase-producer bacteria isolated from compost fertilizer, we decided to conduct such an experiment. In this research a *Pseudomonas* strain was applied to treat oily wastewater whose removing oil capability was much more than that in other researches. Particularly interesting is the residual oil that oily wastewater contains in variable quantities, thus making this waste a potentially suitable growth culture for lipolytic bacteria. The interest of this study is to show that isolated *Pseudomonas* sp can be a strong and appropriate strain for bioaugmentation of aerobic treatment of oily wastewaters with high oil levels. In addition, optimal conditions of the degradation process were identified and proposed.

## Materials and methods

### Isolation and Inoculums preparation

Oil degrading bacteria were isolated from different locations included (1) hot spring, (2) oil wastewater treatment system, (3) refinery and (4) compost fertilizer. A 10-mL sample of the oily wastewater (or supernatant derived from 5 gr compost fertilizer) was added to an Erlenmeyer flask containing 100 mL of olive oil 2% and KH_2_PO_4_ 0.2% and ammonium chloride 0.4% (OPY), at 35°C and shaked (100 rpm) for 48 h. Samples were serially diluted, plated onto tween 80 agar and incubated at 35°C for 72 h. Its lipase activity was distinguished in Tween 80 culture [17], which composed of peptone (1%), Tween 80 (1%), sodium chloride (0.5%), calcium chloride (0.1%), sodium chloride (0.5%), calcium chloride (0.1%) and agar (1.5%). The bacteria had a white halo around the colony. The strain was distinguished using biochemical tests and the bacteria's morphological characteristics [20]. The bacteria were kept on the YPA culture (peptone, 0.5%, yeast extract, 0.3% and agar 1.5%) at 4°C temperature.

### Enzyme assay

The bacteria's lipase activity was measured using polyvinyl alcohol and olive oil emulsion as substrate at 35°C. Cells were separated from the cultivation medium by centrifugation at 6000 rpm for 30 min and the supernatant was used as the source of extracellular lipase. Oil emulsion prepared by mixing 25 ml of olive oil and 75 ml of poly vinyl alcohol 2% solution in homogenizer for 3 min at 5000 rpm. The reaction mixture containing 5 ml of olive oil emulsion, 4 ml of 0.1 mol Tris buffer (pH = 8), 1 ml of 0.1 mol CaCl_2_ and 1 ml of the culture supernatant was incubated at 37°C for 20 min through orbital shaking. The emulsion was immediately disrupted after incubation by the addition of 15 ml of acetone–ethanol mixture (1:1 v/v), and the liberated free fatty acids were titrated with 0.05N NaOH. One unit of lipase activity was defined as the amount of enzyme required to release one micromole of fatty acid per minute under the test conditions [21].

### Biomass concentration

The cell mass was calculated through measuring the amount of culture's absorbance in wavelength of 600 nm (OD_600_) by the spectrophotometer. Then it was calculated based on g/l by the cell mass and the absorbance amount [22]. Cell concentration was equal 0.48 OD_600_. Three factors indicated the survival the Pseudomonas in synthetic and real wastewater: 1) Survival of Pseudomonas was investigated using re-culture (subculture) of samples after 44 hours. 2) Also, no bacteria growth was observed in the control sample (without Pseudomonas). 3) The medium color changes from clear to milky color indicated the viability of the Pseudomonas.

### Synthetic and real wastewater

The wastewater under study was synthetically produced and contained distilled water, ammonium nitrate (4 g/l), sodium chloride (0.5 g/l) and KH_2_PO_4_ (2 g/l). Olive oil with various concentrations (1.5-22 g/l) was added too. PYA-slant cultures were used as the inoculums for precultures. Incubations were carried out at 30°C for 24 h under shaking (100 rpm) in Erlenmeyer flasks (250 ml) containing 100 ml of PY medium. Then as much as five percent of the pre-cultures contained *Pseudomonas* sp. were added to the synthetic wastewater and the amount of oil removal was measured at 30°C and in the shaker at the speed of 100 rpm in various times. All experiments were repeated. The effect of temperature on oil degradation by bacteria was studied. Strains degraded initial concentration of 8.4 g/l oil wastewater with pH 7.0, 100 rpm/min for 44 h at a temperature range from 15 to 40°C. Also, the real wastewater used in this study was prepared by Behshahr Oil Factory-Tehran. To assure prevention from activity of the other intervening biological agents, the real wastewater of the factory was sterilized using autoclave. Table 1 shows its characteristics.

**Table 1:** Characteristics of oily wastewater supported from Behshahr factory

**Concentration (g/l)**	**Parameter**
26000 ± 500	COD
20 ± 5	Ammoniac
300 ± 10	Phosphate
5500 ± 100	Oil and grease
8.6	pH
30°C	Temperature

### Oil concentration measurement

Oil concentration measurement was conducted using the standard method of gravimetric, during which the oil was extracted three times using the Hexane and transferred to a distilling flask with a specified weight. Then the Hexane was evaporated at 85°C temperature, and then the flask was reweighted. The oil concentration (g/l) was calculated through dividing the first and the second weight difference by the sample volume [23].

## Results and discussion

The ability of 3 strains, which presented the highest lipolytic activity or predominated to growth and degrade an oily wastewater medium, was studied. The selected strains were identified as belonging to the *Flavobacterium (oily wastewater)*, Pseudomonas (compost), and Acinetobacter (refinery) genera. The bacteria originating from compost showed highest removal of oil. Oil removal efficiencies were 30, 47 and 95% by *Flavobacterium,* Acinetobacter and Pseudomonas, respectively. The strain which belonged to a special kind of *Pseudomonas* sp. was applied for the next stages. Lipase activity of the strain was obtained 1.7-2.2 ± 0.1 U/ml in medium supplemented with 2% olive oil and in 21–68 hours after inoculation. All the Erlenmeyer flasks were incubated at 30°C temperature and oil removal rate was monitored in 24 to140 hours. The effect of different nitrogen resources is among the parameters which must be studied for degrading oil biological combinations. This has been usually ignored in similar studies. Seven materials (each sample was 4 g/l) were used as the nitrogen resource in the synthetic wastewater.

**Figure 1:**
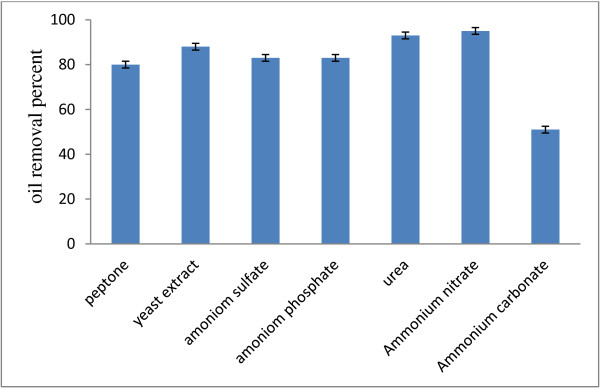
Oil removal percent (8.5 g/l) in 44 hours using various materials as nitrogen resource.

As you see in the Figure 1, the maximum removal yield was achieved in the retention time of 44 hours and with the olive oil's concentration of 8.5 g/l; while ammonium nitrate and urea were used as nitrogen resources and the minimum yield achieved with ammonium carbonate. The removal measured amounts for ammonium nitrate and urea were 95 ± 3 and 93 ± 2.5 percent, respectively. The important point is the amount of the treated wastewater's pH which reduces from 7 to 5 after 68 hours. This reduction may be the result of the fatty acid production through the oil decomposition or the sulfuric acid production. Oily wastewater is usually in need for adding nitrogen during the biological treatment. They face nitrogen shortage, because of containing high amounts of carbon. Therefore, it is necessary to select an appropriate nitrogen resource for their treatment. Of course, a mineral resource is preferable, because the organic nitrogen itself increases the wastewater's organic materials. Therefore, ammonium nitrate is a proper nitrogen resource for the wastewater treatment. In fact, the interactions between pH and nitrogen source showed that the increase of the pH increased oil degradation. In the same study by Brozzoli et al. Oily wastewater supplementation with 2.4 g/l NH4Cl led to an enzyme activity of about 10 U/ml. The addition of malt extract and supplements containing organic N (e.g., peptone, yeast extract) did not affect the enzyme production [24]. Vanot et al. suggested that pH 5.5 was suitable for lipase production by Penicillium. The lipase activity was positively affected by the high concentration of (NH_4_)_2_SO_4_. Similar results were observed concerning the role of ammonium nitrate on lipase production with Geotrichum [22]. By contrast, Annibale et al. found that the same enzyme activity of Penicillium was highest when the fungus was cultivated on Oily wastewater characterized by high organic loads. Goncalves et al. (2009) applied ammonium chloride as a nitrogen resource in this regard and reported the maximum COD removal through using some kind of yeast 60% [25]. Asses et al. (2009) also used ammonium sulfate as a nitrogen resource [22]. Moreover, ammonia consumption and the sulfuric acid production out of ammonium sulfate can reduce pH. Reduction of pH may hinder and reduce the bacteria’s oil degradation activity. At these studies the effect of other nitrogen resources was not considered.

**Figure 2:**
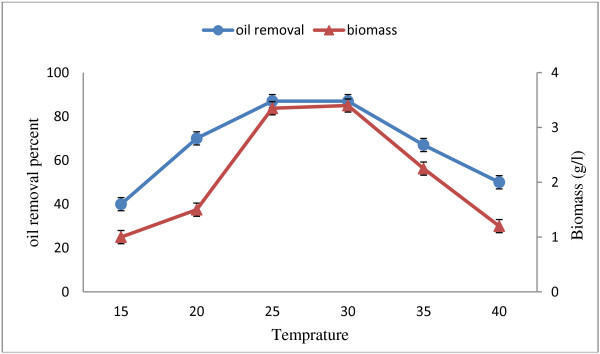
Temperature effect on the oil removal and biomass production with concentration of 12.5 g/l and 24 hours after inoculation.

In oil producing factories, the process temperature is usually selected as 40°C to prevent the oil from changing into solid; therefore the wastewater temperature is about 30-35°C. As a result, it is necessary to use the bacteria which are active at this range. The temperature ranges were considered for the possible variations of temperature in different seasons. This method can also be used for wastewater treatment of other industries (e.g. dairy wastewater at 15-25°C temperatures) with lower temperature. Figure 2 shows the growth of the *Pseudomonas* strain at 15-40°C temperatures. Since the bacteria had appropriate performance at concentration of 8.5 g/l (Figure 1), the concentration increased to 12.5 g/l aiming at considering the bacteria activity at higher concentrations. The results showed that temperature significantly affected oil degradation by *Pseudomonas* sp. The oil removal amount was 40–88 percent in 24 hours retention time. The maximum removal yield for the oil with 12.5 g/l concentration was 88 ± 2 percent achieved at 25-30°C. The findings revealed that this strain growth is at the range of mesophilic. The cell growth (gl^−1^) also demonstrates that the increase of temperature is directly related to cell growth. The maximum growth would be achieved at 30°C and then at over 30°C the growth decrease in 24 hours retention time. This result is confirmed by the oil removal yield. Wu et al.(2009) suggested that the maximum oil removal is achieved using the *Yarovia* yeast at 30°C temperature while at higher temperatures the removal decreases to 40 [16]. In the study of Selva et al., the influence of temperature showed that the lipase production by the *bacillus* was higher at 37°C when compared to those at 27 and 47°C. Walavalkar and Bapat have reported that, the lipase activity of *staphylococcus* was maximum at 37°C [14].

**Figure 3:**
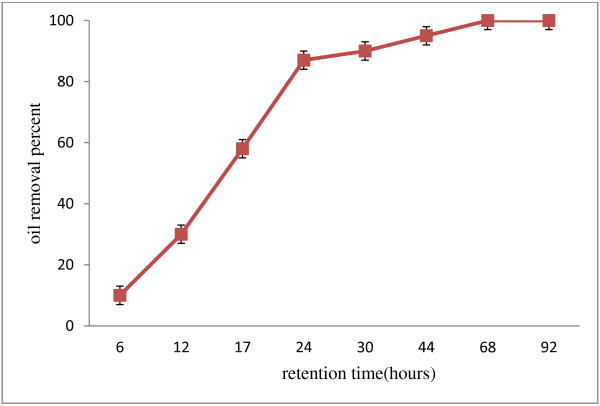
Removal percent of oil with 12.5 g/l concentration in various times and 30°C temperature.

Figure 3 shows the oil removal in different times. The primary oil concentration was 12.5 g/l and incubation temperature was 30°C. The oil concentration was considered for 6–92 hours. The oil degradation rate by strain was significantly different. The highest yield was achieved in 24 hours retention time. Also, lipase activity was 2.2 ± 0.1U/ml in 24 hours which was the highest lipase activity. Oil removal amount was low in 12 primary hours i.e. the cell mass growth is low. However, increase of the cell mass growth increased the amount of the oil removal up to 58 ± 2 and 88 ± 2.5 percent in 17 and 24 hours retention time, respectively. The findings of the Lyliam et al. (2009) showed that applying *Acinetobacter* and *Bacillus* strains provide us with 75% oil removal yield after 50 hours [9]. Moreover, Huiting et al. (2011) achieved 97% yield of the 5 g/l concentration oil removal after 72 hours. They used recombinant yeast which led to 87% oil removal in the early 24 hours [11]. However, we got the higher oil removal in less retention time.

**Figure 4:**
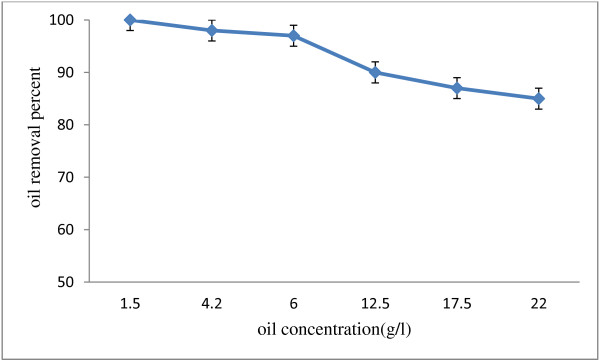
**Various oil concentrations effect on the biological decomposition by *pseudomonas *** **sp. at 44 hours and 30°C temperature.**

Wastewater treatment yield containing various concentrations is shown in Figure 4. In this case and in retention time of 44 hours, the output oil increases with the primary oil concentration increase, while in higher concentration oils, the retention time must be increased. The oil removal amount in concentrations lower than 8.4 g/l was over 95 ± 1.5%. Of course, increase of the oil's concentration to 22 g/l decreases the amount of removal in retention time of 44 hours to 85 ± 2.5%. Moreover, increase of the retention time to 68 hours raised the oil removal to 95 ± 2.5% in 22 g/l concentrations. Wu et al. (2009) studied the fixed *Yarovia* yeast in calcium alginate and found out that increase of the oil concentration from 3 to 5 gl^−1^ leads to oil removal amount reduction from 80% to 45% [14]. However, in our research, the considered *Pseudomonas* strain could remove various concentrations of oil with a high yield and increase of oil concentration to 22 g/l did not change the yield considerably.

**Figure 5:**
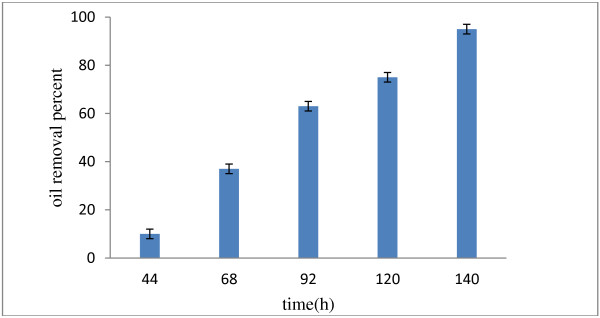
**Removal efficiency of oil by *pseudomonas *** **sp. in real wastewater (Behshahr), various times and 30°C temperature.**

Then at the next stage, we studied the oil removal amounts in the real wastewater of an oil factory. The amount of COD in this wastewater was about 27 g/l, so it was attenuated using distilled water (1:1). The oil removal percent was considered at various times from 44 to 140 hours. The amount of oil removal was 95 ± 2% in retention times of 140 hours (Figure 5). The results showed that the treatment of the real wastewater requires more retention time because of the bacteria adapt to the wastewater conditions. These findings were similar with those in the previous publications (Orapin et al., 2002); although they had reported the amount of oil removal in real wastewater as 95% after 168 hours where the bacillus and *Pseudomonas* strains were applied [26]. Olive-mill wastewater was also investigated for its suitability to serve as a culture for enzyme production by Candida. In this study, Brozzoli et al. showed that OMW might be upgraded as a basis of a medium for the microbial production of an enzyme of commercial interest such as lipase [24]. Although, More work needs to be done to study the selected strains and their use in wastewater treatment process, such as persistence and competence of the inoculated strains with the other microorganisms.

**Figure 6:**
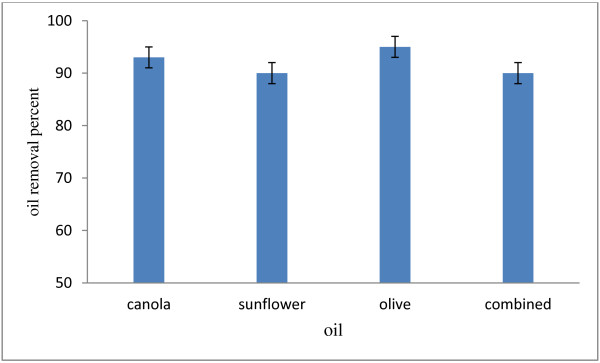
Removal efficiency of different oils with concentration of 12.5 g/l, 44 hours time and 30°C temperature.

Finally olive, canola and sunflower oils as well as a mixture of them were added to the synthetic wastewater and the strain's reaction was studied to assure the capability of this strain in removing all kinds of oil. Figure 6 demonstrated the various oils removal with concentration of 8.5 g/l at about 90-95 ± 2%. Removal percent for the mixed oil which contains olive, canola and sunflower oils in 44 retention time was about 90 ± 1.5 percent. This amount was 95 ± 2% for olive, 90 ± 1.5% for sunflower and 93% ± 2 for canola. Lipase activity was also proportionate to the oil type, 2.2 ± 0.1, 1.1 ± 0.05 and 1.25 ± 0.05, respectively. It means that olive oil is a better stimulator in producing lipase enzyme, as a carbon resource. This finding is in conformity with the other studies in this regard [11]. The study shows that oily wastewaters might be upgraded as a basis of a growth medium for the bacterial production of a lipase enzyme. In fact, significant amounts of this enzyme can be produced on nitrogen-supplemented oily wastewaters based media by *Pseudomonas* sp, thereby enhancing the rates of oil degradation and wastewater treatment.

## Conclusions

The findings of the research revealed that *Pseudomonas* sp. which was isolated from the compost fertilizer can degrade the high concentration of oil. The *Pseudomonas* sp. as a lipase producer is able to remove such oils at mesophilic temperature range in a short retention time; and the increase of the oil concentration and the oil type do not affect the yield. Therefore, this strain may develop the biological treatment in processes of the oily wastewater treatment.

## Competing interests

The authors declare that they have no competing interests.

## Authors’ contribution

All authors have made contribution to the review/finalization of this manuscript and approved the final manuscript.

## References

[B1] Chien L Wen C Joshu C Methods for rapid screening and isolation of bacteria producing acidic lipase: feasibility studies and novel activity assay protocols World J Microbiol Biotechnol 2007 23 633 640 10.1007/s11274-006-9272-8

[B2] Huiting S Lichao Z Lujia Z Bei G Dongzhi W Yaling S Construction of a whole-cell catalyst displaying a fungal lipase for effective treatment of oily wastewaters J Mol Catalys Enzym 2011 71 166 170 10.1016/j.molcatb.2011.04.015

[B3] Jeganaesan J George N Amarjeet B Hydrolytic pretreatment of oily wastewater by immobilized lipase J Hazard Mater 2007 145 127 135 10.1016/j.jhazmat.2006.11.004 17166661

[B4] Izanloo H Mesdaghinia A Nabizadeh R Nasseri S Naddafi K Mahvi AH Nazmara S Effect of organic loading on the performance of aerated submerged fixed-film 85 reactor (ASFFR) for crude oil-containing wastewater treatment Iran J Environ Health Sci Eng 2010 6 85 90

[B5] Salahi A Mohammadi T Rekabdar F Mahdavi H Reverse osmosis of refinery oily wastewater effluents Iran J Environ Health Sci Eng 2010 7 413 422

[B6] Ghidossi R Veyret D Scotto J Jalabert T Moulin P Ferry oily wastewater treatment, Separation Purific Tech 2009 64 296 303 10.1016/j.seppur.2008.10.013

[B7] Akanbi T Kamaruzaman L Abu Bakar F Sheikh A Radu S Abdul M Highly thermo stable extracellular lipase-producing *Bacillus* strain isolated from a Malaysian hot spring and identified using 16SrRNA gene sequencing Internet Food Res J 2010 17 45 53

[B8] Bayoumi R Ellouboudey S Sidkey N Abd R Production, Purification and Characterization of Thermoalkalophilic Lipase for Application in Bio-detergent Industry J Appl Scie Res 2007 3 1752 1765

[B9] Li G Jian X Xinjun L Zuo-Zhen L Optimization of Serratia marcescens lipase production for enantioselective hydrolysis of phenylglycidic acid ester J Ind Microbiol Biotechnol 2004 31 525 530 10.1007/s10295-004-0182-1 15549608

[B10] Lyliam L Mario D Ana L Guzmán I Leticia V Francisco C Isolation and selection of native microorganisms for the aerobic treatment of simulated dairy wastewaters Biores Technol 2009 100 1762 1766 10.1016/j.biortech.2008.09.056 19010666

[B11] Mafakher L Mirbagheri M Darvishi F Nahvi I Zarkesh H Emtiazi G Isolation of lipase and citric acid producing yeasts from agroindustrial wastewater New Biotech 2011 27 337 340 10.1016/j.nbt.2010.04.006 20450991

[B12] Nara G André P Daniela R Francieli A Renata V Jeferson L Isolation and Screening of Lipase-Producing Fungi with Hydrolytic Activity Food Bioprocess Technol 2011 4 578 586 10.1007/s11947-008-0176-5

[B13] Namita G Vikram S Rani G Alkaline lipase from a novel strain Burkholderia multivorans: Statistical medium optimization and production in a bioreactor Process Biochem 2007 42 518 526 10.1016/j.procbio.2006.10.006

[B14] Selva T Palavesam A Immanvel G Isolation and characterization of lipase-producing *Bacillus* strains from oil mill waste African J Biotech 2008 7 2728 2735

[B15] Hasanuzzaman M Kathryn M Szilvia M Naoki M Isolation, Identification, and Characterization of a Novel, Oil-Degrading Bacterium, Pseudomonas aeruginosa Current Microb 2004 49 108 114 10.1007/s00284-004-4267-x 15297915

[B16] Wu L Ge G Wan J Biodegradation of oil wastewater by free and immobilized *Yarrowia lipolytica* W29 J Environ Scie 2009 21 237 242 10.1016/S1001-0742(08)62257-3 19402428

[B17] Aliyu S Zahangir A Ismail A Hamzah S Suitability of using palm oil mill effluent as a medium for lipase production, African J Biotechnol 2011 10 2044 2205

[B18] Anna D Biodegradation of volatile CFCs, H-CFCs and VC in compost and marl Waste Manage Res 1998 16 330 341 10.1177/0734242X9801600405

[B19] Ghazifard A Kasra R Etemadi Z Identification of thermophilic and mesophilic bacteria and fungi in Esfahan municipal solid waste compost Waste Manage Res 2001 19 257 261 10.1177/0734242X0101900307 11699859

[B20] Vaziri Bbiochemical tests principles in diagnostic microbiology 1998 Tehran: Amir Kabir publication

[B21] Colen G Gonc R Junqueira A Tasso M Isolation and screening of alkaline lipase-producing fungi from Brazilian savanna soil World J Microbiol Biotechnol 2006 22 881 885 10.1007/s11274-005-9118-9

[B22] Asses N Ayed L Bouallagui H Ben R Gargouri I Hamdi M Use of Geotrichum candidum for olive mill wastewater treatment in submerged and static culture Biores Technol 2009 100 2182 2188 10.1016/j.biortech.2008.10.048 19091553

[B23] APHA, AWWA, WPCF Standard Methods for the Examination of Water and Wastewater 1989 17 Washington DC

[B24] Brozzoli V Crognale S Sampedro I Federici F Annibale A Petruccioli M Assessment of olive-mill wastewater as a growth medium for lipase production by Candida cylindracea in bench-top reactor Biores Technol 2009 100 3395 3402 10.1016/j.biortech.2009.02.022 19303284

[B25] Goncalves C Marlene L Joao P Isabel B Biological treatment of olive mill wastewater by non-conventional yeasts Biores Technol 2009 100 3759 3763 10.1016/j.biortech.2009.01.004 19231162

[B26] Orapin B Achara K Suptawee F Biotreatment of High Fat and Oil Wastewater by Lipase Producing Microorganisms Nat Sci 2002 36 261 267

